# Improving End-User Trust in the Quality of Commercial Probiotic Products

**DOI:** 10.3389/fmicb.2019.00739

**Published:** 2019-04-17

**Authors:** Scott A. Jackson, Jean L. Schoeni, Christina Vegge, Marco Pane, Buffy Stahl, Michael Bradley, Virginia S. Goldman, Pierre Burguière, John B. Atwater, Mary Ellen Sanders

**Affiliations:** ^1^National Institute of Standards and Technology, Gaithersburg, MD, United States; ^2^Eurofins Food Integrity and Innovation, Madison, WI, United States; ^3^Chr. Hansen, Hørsholm, Denmark; ^4^Biolab Research srl, Novara, Italy; ^5^DuPont Nutrition & Health, Madison, WI, United States; ^6^GNC/Nutra Manufacturing, Inc, Greenville, SC, United States; ^7^Department of Dietary Supplements and Herbal Medicines, Science Division, US Pharmacopeial Convention, Rockville, MD, United States; ^8^AMA Research Solutions, Lyon, France; ^9^US Pharmacopeial Convention, Rockville, MD, United States; ^10^International Scientific Association for Probiotics and Prebiotics, Sacramento, CA, United States

**Keywords:** probiotics, USP, quality, standards, probiotic stability, probiotic identity, probiotic enumeration, third-party verification

## Abstract

In a rapidly growing global probiotic market, end-users have difficulty distinguishing between high quality and poor quality products. This ambiguity threatens the trust consumers and healthcare providers have in probiotic products. To address this problem, we recommend that companies undergo third-party evaluations to certify probiotic quality and label accuracy. In order to communicate about product quality to end-users, indication of certification on product labels is helpful, although not all manufacturers choose to use this approach. Herein we discuss: third-party certification, the process of setting standards for identity, purity, and quantification of probiotics; some emerging methodologies useful for quality assessment; and some technical challenges unique to managing quality of live microbial products. This review provides insights of an Expert Panel engaged in this process and aims to update the reader on relevant current scientific methodologies. Establishing validated methodologies for all aspects of quality assessment is an essential component of this process and can be facilitated by established organizations, such as United States Pharmacopeia. Emerging methodologies including whole genome sequencing and flow cytometry are poised to play important roles in these processes.

## Introduction

Probiotics are being actively researched as agents to enhance health and mitigate disease. Excitement in their potential is fueled by emerging science demonstrating that many physiological processes are impacted by the microbes in and on the human body. Probiotics were defined by a consensus panel of experts in 2014 as “live microorganisms that, when administered in adequate amounts, confer a health benefit on the host” (Hill et al., 2014). Health benefits of probiotics have been studied in controlled human trials, which document a diversity of benefits (Sanders et al., 2018).

The population’s growing interest in natural products that support or enhance health, along with the increasing number of well-controlled human studies involving probiotics, have spurred product development and sales in the probiotics sector. The total global retail market for all probiotic products was estimated at ∼$45.6 billion U.S. for the year 2017, with a predicted compound annual growth rate of 7% (2017–2022) (MarketsandMarkets, 2017). One market analysis forecasts that the global market for probiotics as ingredients will grow from ∼$1.71 to ∼$3.56 billion U.S. during 2016 – 2025 (Grand View Research Inc, 2017b) and the probiotic dietary supplements sector, which ranked second only to probiotic foods and beverages, will grow from ∼ $3.3 billion U.S. to ∼ $7.0 billion U.S. (compound annual growth rate ∼7.5%) (Grand View Research Inc, 2017a).

Probiotic products are unique in that they are designed to deliver live microorganisms to the end user to confer a health benefit. Unlike traditional dietary ingredients, live microorganisms represent different challenges in design, development, scale-up, manufacturing, commercialization and life cycle management. Recently, observations of uneven quality in probiotic products have been reported: examples range from products not meeting claimed active counts and incorrect strain identification (Drago et al., 2010; Weese and Martin, 2011; Morovic et al., 2016) to the tragic infant death linked to a fungus (*Rhizopus oryzae*)-contaminated probiotic product (Vallabhaneni et al., 2015). If unaddressed, quality concerns will erode both professional and lay consumer confidence in these products, resulting in decreased usage and sales. Further, maintenance of viability during product storage is a challenge, as numerous environmental factors such as carrier material (including different food matrices), temperature, water activity, and storage time, can impact probiotic survival (Schoeni, 2015).

Quality and safety of probiotic foods and supplements is the responsibility of industry. Therefore, manufacturers can benefit from a transparent means of communicating product quality to consumers via independent assessments. Here, the USP Probiotics EP shares comments and recommendations regarding challenges observed within the industry. The intent of this article is to assist in improving the quality of probiotic products and enhance transparent communications between manufacturers, regulators, and consumers through science-based assessments.

## Third-Party Verification, Certification, and Qualification

Verification, certification or qualification by an independent third-party organization is a process undertaken to provide assurance of quality and to facilitate regulatory compliance of an ingredient or finished product. Critical to this “third-party certification” process is that the certifier remains independent from the manufacturer.

The typical third-party certification includes assessment of manufacturing processes, which must comply with applicable GMP regulations, review of critical documentation, as well as independent testing to verify compliance with certain expressed or implied claims made in labeling. Third-party organizations use methods either supplied by the manufacturer, which may not be publically available, or if available, public standards. In either case, it is important that users of the method perform their own due diligence to ensure that methods are suitable for intended purposes.

The third-party certification process usually includes the following steps, which may differ depending on the certification body:

(1) Application: Most third-party certifications start with submission of an application to a certification body, preferably a body with ISO 17020 and/or 17065 Registration. A confidentiality agreement and feasibility assessment may be undertaken.
(2) Documentation submission: Documentation includes information about facility statistics, such as square footage, number of employees, and product lines. Certain critical documents such as Standard Operating Procedures Index, Analytical Methods Index, Quality Manual Index, Hazard Analysis and Critical Control Point Plan, and Allergen Control Plan may be requested for preliminary review prior to a GMP audit.
(3) GMP audit: The GMP audit comprises a full audit of the quality management systems, including management oversight of quality, facilities and equipment, materials management, production systems, packaging and labeling controls, and laboratory controls.
(4) Documentation review: The documentation review is an in-depth review of critical GMP documents conducted off-site by an individual. These documents include: master batch record, executed batch records, in-process testing records, executed packaging batch records, stability substantiation, and finished product testing records.
(5) Verification of Certificate of Analysis: Prior to engaging in any product testing, all testing methods must be verified (fit for purpose) and harmonized along the supply chain. This includes methods needed for Certificate of Analysis, including measurement of identity, strength, purity, composition and limits on contaminants. For probiotic companies, microbial enumeration methods are especially challenging, as even subtle differences in sample preparation, diluent buffers, and microbiological media can have a significant impact on the recovery of viable organisms.
(6) Corrective actions: The certification body will issue a list of needed corrective or preventative actions. Depending on the nature of the corrective actions, the certification body may require resolution of the corrective action prior to issuing formal certification.
(7) Certification: The last step in the process is formal issuance of the certification for the product. Once a company achieves certification, then the certification body will establish criteria for recertification. Recertification usually includes the same basic steps; however, the extent of the due diligence may be reduced after the certified company establishes a history of good compliance.

It should be noted that these steps to certification are not always sequential, and can be executed by the independent third-party certifier concurrently.

Third-party certification of products provides a high degree of assurance that the products introduced into the market are not adulterated or misbranded. Some companies choose to certify for brand protection, while others choose to certify for brand promotion, which may differentiate their products from other non-certified products in the market.

Several organizations provide third-party certifications. These organizations differ with regard to scope of assessments, for-profit status, and issuance of a seal for certified products. Table 1 summarizes information on third-party certification organizations, which currently certify probiotic dietary supplements.

**Table 1:** Descriptions of organizations offering third-party certification services for probiotics.

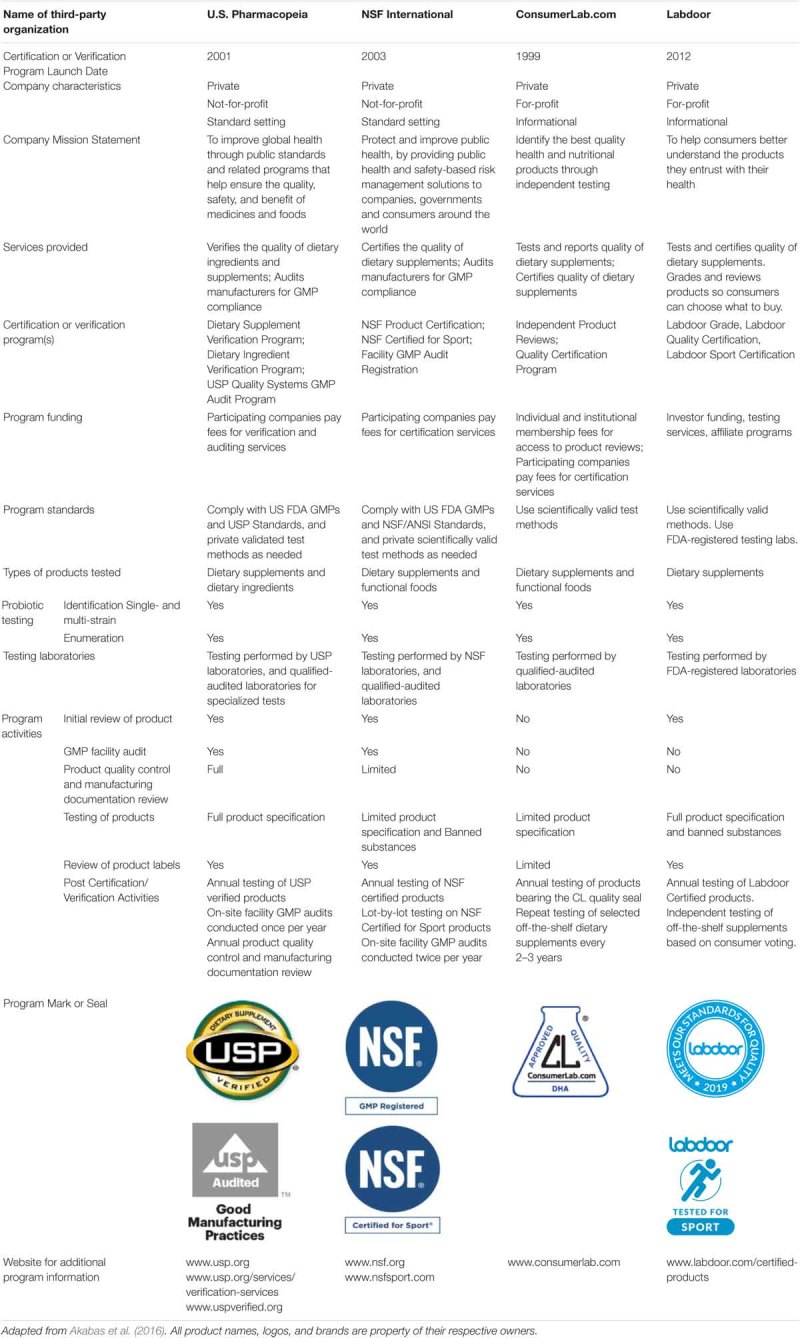

Quality seals from these organizations can help consumers recognize products that have been independently tested and shown to meet their ingredient label claims, and for some seals, that the product was manufactured under stringent conditions. One certification organization, the European Scientific League for Probiotics, is focused on European products. This non-profit organization was founded in 2011 to provide a quality seal to probiotic products that are properly labeled with the strain designations and meet claims of viability throughout their shelf life.

## Quality Standards

In the context of quality control for dietary supplements, a standard is a document that provides requirements, specifications, guidelines or characteristics that can be used consistently to ensure that materials, products, processes and services are fit for their purpose. Such standards are established or required by government and non-governmental agencies, such as FDA, the European Food Safety Authority, Codex Alimentarius, and others, and are applicable for testing and labeling of raw materials in order to establish quality. A pharmacopeia is a compilation of standards. Most pharmacopeias contain publicly available standards, comprising a list of tests, test methods and acceptance criteria. Together these make up the specifications for an ingredient or product. These specifications define identity, purity, content (strength/composition) and quality in terms of performance or other attributes. Pharmacopeial standards, as opposed to internal company standards, provide a common basis of quality for medicines, food ingredients and dietary supplements shared across different manufacturers. When adopted by manufacturers, these standards contribute to public health by decreasing the likelihood of contamination, adulteration and improper composition of commercial products.

There are several public pharmacopeial standard setting bodies such as national and regional government pharmacopeias in Europe, the United Kingdom and Japan. Also, the World Health Organization develops standards. AOAC International does not develop pharmacopeial product standards, but develops consensus-based public analytical testing standards. One advantage of public standards is that they provide a common understanding among stakeholders regarding validated and accepted testing methods applicable to quality testing throughout the production process.

The USP is a non-governmental, non-profit scientific organization founded in 1820 dedicated to improving public health through development of quality standards for drugs, dietary supplements and foods. USP is recognized by the FDA, which enforces USP standards in the pharmaceutical industry. USP depends on expert volunteers to provide scientific input, review, and approve the standards that are developed. Numerous Expert Committees support the different types of products. Probiotics fall under the purview of the Non-Botanical Dietary Supplements Expert Committee, which is advised by an EP comprising probiotic experts.

At USP, standards for individual ingredients or finished products are called monographs. Monographs are initiated by a sponsor or donor interested in a quality standard for its product. The sponsor provides validated analytical methods and acceptance criteria for identification (in the case of probiotics, this identity information is provided to the strain level), purity, composition and stability (reflected in viability measurements in the case of probiotics). Using this information, USP independently develops the standard. Analytical methods are validated in multiple laboratories to ensure the methods are robust. After USP review, standards are reviewed by Expert Committee members and then published in the Pharmacopeial Forum (PF). The PF is a free resource that allows opportunity for public comment on all aspects of the monograph. After the comment period (90 days), the monograph and any comments received are sent for review by the expert committee, revised as needed, and approved monographs are published in the USP-National Formulary as official. USP standards undergo continuous revision as stakeholders suggest improvements in methods or when issues with contaminants or adulteration arise.

## Probiotic Product Labels

Probiotics, being composed of live microbes, have label requirements that are somewhat different from other foods and supplements. Guidelines issued by the Joint Food and Agriculture Organization of the United Nations and World Health Organization in 2002 (FAO/WHO, 2002) and later by the International Probiotics Association in partnership with the Council for Responsible Nutrition (Council for Responsible Nutrition and International Probiotics Association, 2017), reinforce the importance of communicating specific information on a probiotic product label. The stipulated information may not be required by national regulations, but there is scientific agreement that such information is important to enable consumers to understand what they are buying. The information includes:

• Genus and species names, which should adhere to current scientifically valid nomenclature.
• Strain designations for each strain in the product. Designations used should enable tracking of the strain to entries in strain depositories and linking to published studies.
• Statement of quantity (using CFU or other validated measure) of live/active microorganisms through the use-by date.
• Use-by date.
• Statement of benefit is not required, but if present must be supported by a human study showing the benefit at the dose delivered in the product.
• Proper storage conditions.
• Company contact information.

## Identity

A fundamental component of the probiotic monograph process is clear identity of the probiotic strain under consideration. Further, the ability to differentiate a probiotic strain that is the subject of a monograph from similar strains submitted to the process is essential.

### Taxonomy

Probiotic strains must be identified by genus and species names based on current scientifically valid nomenclature, including a subspecies when applicable. High-quality, full-length 16S gene sequencing is usually a suitable method for determining species and subspecies. Supporting information for a probiotic monograph should include alignment of the full length 16S rDNA sequence of strain to the type strain of the species/subspecies of interest to demonstrate correct taxonomic classification.

The probiotic field is poised for a significant upheaval in the taxonomy of *Lactobacillus*, an important probiotic genus. The *Lactobacillus* genus is polyphyletic and characterized by unusually high phenotypic and genotypic diversity, which does not conform to taxonomic conventions. Comparative genomic analysis, which revealed 10 robust phylogroups, is the basis for reclassification (Salvetti et al., 2018). The genus could be split into as many as 23 new genera, but species names will remain unchanged. Many traditional probiotic species such as *L. plantarum, L. reuteri, L. rhamnosus*, and *L. casei* will likely no longer be members of the *Lactobacillus* genus. As of this writing, this process is still underway and no confirmed changes are decided.

Nomenclature changes have frequently affected species used as probiotics. The well-studied probiotic strain, *Lactobacillus rhanmosus* GG, was initially identified as *L. acidophilus*, briefly as *L. casei* and currently as *L. rhamnosus*. The probiotic species commonly referred to as *Bifidobacterium lactis* is correctly designated *Bifidobacterium animalis* subsp. *lactis*. *Bifidobacterium infantis* is now recognized as a subspecies of *Bifidobacterium longum*. Commercial product labels and communications should comply with current nomenclature (Parte, 2018).

### Strain Identity

According to the first edition of *Bergey’s Manual of Systematic Bacteriology* (Staley and Krieg, 1984) “a strain is made-up of the descendants of a single isolation in pure culture and usually is made up of a succession of cultures ultimately derived from an initial single colony.” Furthermore, a well-defined probiotic strain could be considered a genetically unique live microorganism that is essentially clonal in nature. This definition, however, does not resolve the questions of how many genetic differences are reasonable to still be considered unique and what is the definition of ‘essentially clonal.’

### Genetic Drift

Recent research into genetic drift within bacterial strains strongly suggests that probiotic manufacturers should address the risk of genetic drift in their industrial processes. In this context, drift can be defined as divergence via DNA mutation of a bacterial strain over time. DNA mutation generally occurs at a constant low rate unless selective pressure is applied. There are landmark studies on genetic drift in organisms such as *Escherichia coli* (Lenski et al., 2003), but few studies on genetic drift in probiotics. Assurance of genetic stability of a probiotic strain will preserve confidence in the documented efficacy studies, although genetic drift is less of a concern with regard to safety (Sanders et al., 2014). A study reported a strain of *L. rhamnosus* (GG) that exhibited multiple genotypes in consumer products (Sybesma et al., 2013). It is unclear if the multiple genotypes were truly a result of genetic drift, as reported, or a result of a mixed population in the original seed. Strict process control during scale up and growth is imperative to ensure the fewest number of generations as possible from the mother seed, thus reducing potential for genetic drift. Guidelines for methods of storage of microbial cultures, and long-term stability of microorganism should be used to reduce risk of variation from within the population. Biological resource centers such as the American Type Culture Collection have put forward such guidelines for strain preservation and avoidance of contamination and deterioration with optimized protocols that reduce the risk of drift during growth and preservation of microorganisms (European Consortium of Microbial Resources Centres, 2018). Ultimately, the measure of genetic drift during strain propagation via deep-sequencing post-production should be conducted. Further, it is prudent to confirm the whole genome sequence when embarking on new investigational research for a probiotic strain prior to manufacturing the material for study.

### Whole Genome Sequencing

Rapid and affordable DNA sequencing technologies, under the broad category of NGS technologies, have been widely adopted by laboratories around the world to conduct WGS. There are currently four major technologies that dominate the field of genomic sequencing. These four technologies are Illumina, Oxford Nanopore, PacBio, and Ion Torrent. These technologies differ based on how the DNA sequence is “read”, and their relative performance is generally assessed by considering three metrics: sequence quality, read length, and cost (Table 2).

**Table 2:** Comparison of performance metrics for the four major next-generation sequencing technologies.

	PacBio	Illumina	Ion Torrent	Oxford NanoPore
Error Rate	High	Low	Low	High
Read Length	Long	Short	Short	Long
Cost	High	Low	Low	High

*To generate a high quality whole genome sequence, a combination of methodologies assuring both low error rate and long read length must be used. The ideal technology would provide long read lengths and low error rates, but such a technology does not yet exist. Hybrid (orthogonal) sequencing technologies must be employed in order to generate a high-quality complete (closed) genome sequence. Error rate is a reflection of sequence quality. Longer read lengths facilitate complete genome assemblies. Long read lengths are typically 10’s of kilobases. Short read lengths are typically 100’s of bases. High error rates are estimated to be greater than 1%, whereas low error rates are less than 0.1%.*

At the time of this writing, none of the current NGS technologies can produce a complete genome sequence. Rather, raw data generated by an NGS instrument is in the form of relatively short sequences (reads) that represent small fragments of the organism’s genome. Read lengths typically range from 100 bases to 10’s of kilobases, depending on the technology employed. Only by reading millions of random genome fragments, in parallel, do these NGS technologies provide coverage of an entire genome. Raw sequence data are assembled *de novo* to produce a complete genome assembly (here a “complete genome assembly” refers to an assembly that contains no gaps in the sequence). To achieve a complete genome assembly, it is necessary to utilize a long-read DNA sequencing technology to resolve regions of the genome that are redundant (repeats). However, to manage high error rates of long-read sequencing technologies, an orthogonal technology that does not suffer from a high error rate (e.g., Illumina) should also be employed. These data generated from higher fidelity sequencing technologies can be used to correct the long-read, error-prone sequence data to yield a high quality complete genome sequence. Open-source genome assemblers such as Canu (Koren et al., 2017), SPAdes, and Pilon (Walker et al., 2014) have been developed for doing long-read assemblies and error correcting with short, accurate reads.

### Comparative Genomic Analysis to Demonstrate Strain Uniqueness

Whole genome sequencing should be performed on each probiotic strain to confirm its identity. This should be performed on cells derived directly from the master cell bank to limit genetic drift during the culturing process. This WGS need only to be performed once on the master cells, and then can be used as a reference genome sequence for subsequent DNA-based analytical methods (such as PCR). Here we describe how these data might be used to identify and differentiate individual strains.

Once high-quality, complete genome assemblies have been generated, these reference genome sequences can be used to demonstrate a strain’s uniqueness. This can be done using a number of different open-source analysis tools. An open-source, easy-to-use tool that has been used successfully to align and compare genomes at both the structural level and the nucleotide level is known as Mauve (Darling et al., 2004), although other open-source genome aligners are available, such as MuMmer (Marçais et al., 2018). Mauve is capable of aligning two genomes in a matter of minutes and reports single-nucleotide-level differences between the genomes as well as structural rearrangements. Mauve can also be used to align multiple genomes, although compute time scales cubically with the number of genomes being aligned.

**FIGURE 1:**
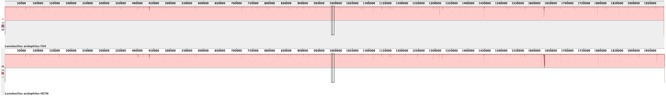
Authors illustrate alignment of two publicly available genomes, which can be useful to determine differences among different strains. Mauve genome alignment of *Lactobacillus acidophilus* strains FSI4 and NCFM. Pink bars represent the entire lengths of each (∼2 Mbase) genome. The red hashes seen across the top of each genome represent the presence of single nucleotide polymorphisms (SNPs). There were 270 SNPs identified that could differentiate these two strains.

As a demonstration, we used Mauve to align the genomes of two strains of *Lactobacillus acidophilus* whose genomes sequences were publicly available at NCBI’s GenBank. The Mauve alignment results indicated that the genomes of *L. acidophilus* strains FSI4 (NZ_CP010432.1) and NCFM (NC_006814.3) were highly contiguous but were also easily differentiated by the presence of 270 SNPs that spanned the entire length of the genome (Figure 1).

**FIGURE 2:**
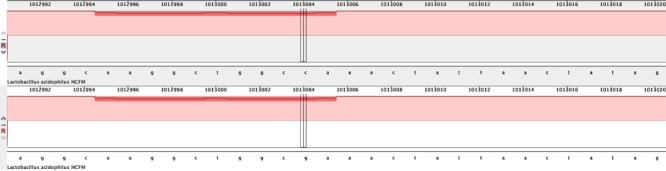
Mauve alignment of *Lactobacillus acidophilus* strains NCFM and NCFM-SNP to illustrate how a single nucleotide polymorphism (SNP) (C->G) can be identified.

Mauve can also be used to differentiate two strains that differ by a single SNP. To demonstrate this, we edited the *L. acidophilus* NCFM genome *in silico* by changing a single nucleotide at position 1013004 from C to G. This edited genome, named NCFM-SNP, was aligned with the NCFM genome using the default Mauve (progressiveMAUVE) parameters. The results revealed the single SNP that we introduced (Figure 2). This demonstrates the ability to detect single SNP differences among different strains.

High quality whole genome sequences provide resolution to the single base pair for identifying and differentiating strains of probiotics. Because this level of differentiation is technologically feasible, it is theoretically possible to conclude that two strains are distinct strains if a single nucleotide difference is detected, even if no phenotypic differences are identified. The ability to determine if two strains are the same or different is more than an academic exercise in the context of establishing identity for a public probiotic monograph. Identical strains, even if submitted under different names, would fall under the same monograph. If there is no genotypic or phenotypic means to distinguish the strains, they would be considered identical. There is no consensus within the scientific community on the number of base differences that would define a new strain. The opinion of many on the EP is that a single base pair cutoff is logical and the least arbitrary in this context. Alternatively, strains could be defined by a combination of genotypic and phenotypic traits.

Considering the value of a high quality whole genome sequence for identity and safety assessment of a probiotic strain, the EP considers WGS an essential part of a probiotic strain’s portfolio. At the time of this writing, a high quality complete bacterial genome can be obtained for ∼$4000 USD through commercial services. This affordable price includes ∼100x genome coverage using two orthogonal sequencing technologies that generate long and short reads, enabling the hybrid assembly approach described above. Surely such an investment should be seen as essential before any strain is evaluated in a human clinical trial.

If WGS is to become the industry standard for strain identity, manufacturers must also be willing to provide open access to their reference genome sequence data, and their strains should be available for non-commercial purposes from an international culture collection. Sharing will enable manufacturers to perform comparisons between their strain and strains from other manufacturers. These comparisons can be performed either *in silico* using the WGS data or on DNA isolated from the strain. Further, in principle, the availability of commercial strains for non-commercial use would allow independent scientists to repeat published studies, an important aspect of the scientific process. If a repeated trial fails to confirm previous findings, the availability of the WGS would enable researchers to confirm that the same strain was assessed in both trials.

Once a reference genome sequence is available, it can be used to develop strain-specific PCR primers, and these can be used to perform rapid and affordable PCR-based strain identification assays. It is also possible to use WGS as an alternative to PCR to demonstrate the identity of a manufactured lot, by generating WGS data from DNA extracted directly from the manufactured product. These WGS data need not meet the criteria described above for generating a high quality complete genome sequence. Instead, relatively low coverage WGS data generated using a rapid and affordable sequencing technology can be quickly mapped to the reference genome sequence. In doing so, manufacturers can be confident that the manufactured product contains the correct strains.

This same WGS data can also be used as a measure of product purity. In the case that a manufactured product becomes contaminated with another microorganism, the WGS data would contain genome sequence data from the contaminating organisms, which could be identified by cross-referencing the WGS data with a database of reference genomes (e.g., NCBI’s GenBank). The sensitivity of this assay is directly dependent on the amount of sequence data (number of reads) generated and the relative abundance of the contaminant. Finally, these same WGS data can be used to measure relative abundance of individual strains contained in a multi-strain, blended product. Lot-to-lot variations in the relative abundance of individual strains can be determined using this method. Because of these many advantages to NGS-based assays, they surely will be adapted to the GMP setting, although currently these methods are not being used in this manner.

An emerging area is use of WGS-based assays to measure viability. These techniques exploit the presumption that cells with compromised membranes are “dead.” DNA-reactive chemicals, impermeable to intact cell membranes, are used to permeate cells with compromised membranes. Once inside these “dead” cells, the chemicals react with the DNA and render it incompetent for sequencing (Nocker et al., 2007). These methods are still under development, but in the future WGS-based assays may be suitable for assessing stability and viability of probiotic products.

## Quantification

Probiotics by definition must be live microorganisms. Therefore, quantification using a measure of viability is essential. It is the responsibility of the manufacturer to ensure sufficient product stability throughout production and distribution in order to ensure the labeled quantity of viable probiotic cells until the expiration date. In most cases, this is accomplished using a combination of technologies that sustain microbial viability and formulating with overages that will allow for some probiotic death without the total count dropping below the labeled potency. Important factors in probiotic stability include matrix design, container/closure system, water activity of final formulation, storage temperature, handling and distribution logistics, as all can have a major impact on product viability and shelf life. Unlike conventional dietary ingredients, reliable accelerated stability testing methods to extrapolate shelf life are not feasible for probiotics. Refrigeration, low oxygen, and low water activity can significantly extend shelf life.

### Microbial Viability

Traditionally, bacterial viability is taken as the capacity of a cell to replicate to detectable levels, either as a colony on agar or by turbidity in broth. However, bacterial viability can also be interpreted as the presence of an intact cytoplasmic membrane, synthesis of proteins and nucleic acids, metabolism, and eventually multiplication (Breeuwer and Abee, 2000). Nevertheless, culturability is still considered the most unique parameter of microbial viability. However, this interpretation is not without challenges. Apart from the fact that not all bacterial phyla are actually culturable by traditional methods (Davey, 2011), bacteria can also rest in states of dormancy, which does not involve active replication (Lennon and Jones, 2011). A reversible condition of dormancy is well described for sporulating bacteria and, to some extent, for toxin-antitoxin driven persister cells of *E. coli*, where revival from dormancy can be induced by favorable changes in environmental conditions (Rittershaus et al., 2013). In contrast, a less well-defined state of dormancy is the VBNC state, a description which is often applied to bacterial populations displaying metabolic activity but loss of culturability (Lennon and Jones, 2011). The VBNC state remains controversial as there is little scientific evidence of revival from this state to active replication and proliferation. Metabolically active VBNC cells may recover from sublethal injuries and thus become viable under optimal conditions, e.g., in contact with the enteric system, but these cells may also be too injured to proliferate even under optimal conditions (Barer, 1997).

Viable but non-culturable cells can frequently be observed in probiotic products due to numerous unavoidable stressful processes that probiotic cultures undergo during industrial production. Fermentation, biomass concentration, cryopreservation, lyophilization, powder grinding, and storage represent processes that can, even when optimized, drive cells to enter in a VBNC state (Lahtinen et al., 2006; El Arbi et al., 2011). Modern technology allows microbial capability to be assessed beyond culturability, by measuring membrane integrity (as discussed for WGS methods, above) or metabolic activity of individual cells. However, in practice there is a significant difference between measures of metabolic activity and culturability, especially when quantifying preserved cells over the course of time (Hoefman et al., 2012). Virtually all publications dealing with efficacy of probiotics measure doses by CFU. Therefore, any alternative method of measure of viability for a commercial product claiming benefits substantiated through clinical trials will need to establish equivalency with potency measures in the clinical trials. Given the highly diverse physiology of different probiotic strains, it is very likely that there is significant species and strain variation in this matter.

### Plate Count

Bacterial enumeration by plate count methods measures bacterial cells able to proliferate into detectable colonies on agar media and thus the results are specified in CFU. The practice of enumerating bacteria by CFU has been used since the late 19th century and is still the gold-standard method to quantify bacteria in the probiotic industry. Most recognized standards such as those published by ISO, the IDF, and USP use plate count methods for bacterial enumeration of beneficial bacteria as well as contaminants (International Standards Organisation [ISO], 2006, 2010; United States Pharmacopeia, 2013).

The benefits of plate counts are technical simplicity and ease of implementation. Further, it is clear that a colony results from a viable progenitor, either one or more viable cells in proximity on an agar plate. Therefore, declaration of the quantitative amount of probiotics as CFU per serving is in agreement with the definition of probiotics as *live* microorganisms. In addition, clinical studies investigating the effect and dose-response of probiotics generally use CFUs as a dose measure (Mobini et al., 2017; Ouwehand, 2017). Analyzing and declaring the probiotic content of commercial products in CFUs will therefore match doses with those used in clinical studies, providing a clear link to available evidence of health benefit. This will help consumers and health care professionals make informed choices when choosing the product with the desired properties.

The challenges associated with plate count methods are many. Primarily, no single methodology is applicable to all probiotic organisms because of considerable variability between species and strains in their response to plating procedures (Davis, 2014). There are only a few ISO methods available, e.g., (International Standards Organisation [ISO], 2006, 2010) and numerous internal methods developed by manufacturers to optimize the growth requirements of individual strains exist. Plate count methods are laborious in terms of laboratory workload and sample throughput and are time consuming due to long periods of incubation. There can be considerable technical difficulties in identifying suitable growth conditions for oxygen-sensitive strains or species, which are adapted to the gastrointestinal environment.

The initial sample preparation (rehydration of lyophilized probiotics) is complex and can influence results considerably. Several parameters such as osmotic concentration, pH, buffer capacity, reactive oxygen species, homogenization intensity and aggregates can significantly affect the resulting CFU count (Muller et al., 2010; Champagne et al., 2011). Aggregates or chains of bacterial cells, which may be composed of many individual cells, will give rise to only a single CFU. Finally, bacterial quantification in CFUs will not detect VBNC cells, which could potentially play a role in efficacy heretofore unexamined.

Another challenge to plate count methods is inter-laboratory reproducibility and intra-laboratory repeatability. Due to operator-generated bias, sample preparation complexity, and variation of equipment, the reproducibility can vary considerably among independent analyses. Therefore, a means to reduce variation is needed to obtain accurate CFU counts of probiotic products. Various limits for the critical difference [defined as the 95% confidence interval limit for the absolute difference between two test results obtained under the reproducible conditions (International Standards Organisation [ISO], 1995)] of reproducibility can be found in international standards and national guidelines and range from 0.2 to 0.5 log_10_ (International Standards Organisation [ISO], 2003, 2010). It is up to the individual manufacturers of probiotics to control the repeatability and reproducibility of applied plate count methods; improved reproducibility of methods can avoid the need for excess overage. Method reproducibility can be improved by strain-optimized methods, operator training, qualification and control of equipment and media, while enhanced estimates of the true reproducibility can be obtained by increasing the number of replicates and plates in each analysis.

### Flow Cytometry

Over the last 20 years, multiparametric FC has become a powerful tool in microbiology, particularly in biotechnological processing, food preservation, chemical disinfection processes and pathogen enumeration in clinical and industrial practice. Many attempts have been made to develop rapid and unbiased methods, usually based on the exclusion, uptake or metabolism of colored, fluorescent, or fluorogenic stains, designed to provide information associated with viability. FC measures different structural and functional properties of cells, which enables quantification of cell properties that may correlate with viability not based on traditional reproductive capacity on agar. This approach has the potential to provide insight into the physiology of the probiotic, in addition to its viability (Wilkinson, 2018).

A key advantage of FC is the ability to simultaneously collect multiple data outputs for an individual cell regarding viability, vitality, structural integrity, physiological status, or stage of growth cycle. Flow cytometers offer the advantage of being able to analyze thousands of cells/events per second, depending on sample type and cell concentration in the sample. Bacterial enumeration can be obtained in less than 1 h in triplicate and with high accuracy. For these reasons, FC is emerging as an alternative rapid method for microbial detection, enumeration, and population profiling (Van Nevel et al., 2017).

Flow cytometry detects all the cells in the product (viable, non-viable, VBNC, dormant) and can characterize their physiological state. For example, a study which enumerated probiotics in fermented milk by colony count showed that counts decreased over time (Lahtinen et al., 2006). FC quantification targeting different physiological parameters (esterase activity, membrane integrity, pH gradient) revealed that the probiotics were metabolically active and therefore, still viable (Lahtinen et al., 2006).

The recent approval of FC by ISO shows promise for more widespread acceptance of cytometric profiling as a measure of cell number and viability (International Standards Organisation [ISO] and International Dairy Federation, 2015). Moreover, an international standard was validated for use of FC for fresh cultures of starter cultures, probiotics and fermented products (International Standards Organisation [ISO] and International Dairy Federation, 2015). This particular ISO standard can be applied universally and independently to the species of interest for bacterial enumeration. In this standard, results are expressed results as TFU (Total Fluorescent Units), a unit that reflects the total number of cells regardless of physiological state, and AFU (Active Fluorescent Units), which is a measure of viable cells. In the case of AFU/g obtained for fresh culture preparations, repeatability and reproducibility values were determined as being 0.06 and 0.45 log_10_ while for TFU/g these values were 0.07 and 0.38 log_10_, respectively. These values were defined by collaboration among 15 different laboratories over five countries and nine different FC models (International Standards Organisation [ISO] and International Dairy Federation, 2015). Microbial viability is based on three different physiological parameters: esterase enzymatic activity, membrane integrity, and membrane potential. No significant differences were found for AFU/g obtained using the three protocols for the samples analyzed, indicating a high degree of equivalence for data obtained for the different staining protocols that measure different parameters of bacterial viability (Breeuwer and Abee, 2000). This study did not compare FC results with CFU.

Recently validation of FC was conducted in two different laboratories on three different industrial batches of *L. rhamnosus* GG (ICH Harmonised Tripartite Guideline, 2005). Accuracy, precision (repeatability), intermediate precision (ruggedness), specificity, limit of quantification, linearity, range, robustness demonstrating the validity and robustness of the cytofluorimetric analysis were assessed (Pane et al., 2018). Repeatability of 0.07 and a reproducibility 0.09 log_10_ was demonstrated.

The FAO/WHO probiotic guidelines (Food Agriculture Organization of the United Nations/World Health Organization Working Group Report on Drafting Guidelines for the Evaluation of Probiotics in Food, 2002) recommend that product labels should include information on “minimum viable numbers of each probiotic strain at the end of the shelf-life” but do not stipulate CFUs must be the method used. Indeed, another of this group’s recommendations was “further development of methods (*in vitro* and *in vivo*) to evaluate the functionality and safety of probiotics.” This suggests openness to new approaches for measuring viability. Since the literature reports that non-culturable microorganisms (Salminen et al., 1999) and probiotic-derived factors (Howarth and Wang, 2013) may also confer a health benefit, the ability to quantify such factors may become important in the future.

There is general agreement in the probiotic field that the amount of probiotic bacteria should be expressed in CFU/g or CFU/serving throughout the product shelf-life. The FDA passed regulations stating that all dietary supplements must declare active ingredients by weight, but recently opted for enforcement discretion allowing probiotic products to be labeled also using CFU. In guidance relating to clinical trials for live biotherapeutic products, FDA stated that potency of live microbial products is generally measured in CFU (United States Food and Drug Administration, 2016). At the same time, FDA recognizes that other methods have the potential to accurately and efficiently quantify the number of viable cells, and innovation should be allowed (United States Food and Drug Administration, 2018a,b). However, until FC is used to quantify potency of probiotic interventions in clinical trials, a link must be made between AFU or TFU and CFU if FC is to replace CFU for quantifying potency. Generally in freshly made probiotic culture the correlation of CFU with AFU is close to 1:1 (Pane internal communication), but this relationship does not hold for probiotics over the course of shelf-life. As probiotics age, their ability to form a CFU decreases more quickly than their ability to be counted as AFU or TFU. Thus, assessing viability by FC will likely give higher counts at the end of shelf-life than culture methods, which could translate into an advantage for manufacturers.

## Single Strain vs. Multiple Strain Products

For products containing multiple strains, enumeration is a complex endeavor. A common approach is to determine total microbial count, which does not distinguish among different strains in the product. Alternatively, in some cases selective plating methods targeted toward each strain in the blend can be used. Selective enumeration employs culture media with specific nutrients, conditions or selective ingredients to select for the desired microorganism (Charteris et al., 1997). A recent study published the development of a chromogenic culture medium to differentiate and enumerate several species of lactic acid bacteria (Galat et al., 2016). Few public standards are available for this purpose, although methods exist for the selective enumeration of *L. acidophilus* (International Standards Organisation [ISO], 2006) and *Bifidobacterium* spp. using antibiotics as selective agents (International Standards Organisation [ISO], 2010). These methods present the same limitations as other classical microbiology protocols (Kell et al., 1998; Lahtinen et al., 2005; Oliver, 2005). Moreover, some methods are only selective at the genus level (Ashraf and Shah, 2011). Specific distinction among different species of *Bifidobacterium* or *Lactobacillus* is difficult to achieve with selective media, especially if strains are not present at roughly equivalent levels. Methods to selectively enumerate different strains of the same species in a blend do not exist.

In order to overcome classical microbiology limitations, several molecular biology-based approaches have been investigated for specific quantification of viable probiotics: WGS, real-time quantitative PCR and chip-based digital PCR using propidium monoazide or related molecules (Nocker et al., 2006, 2007; Kramer et al., 2009; Hansen et al., 2018). Based on membrane integrity assessment, these methods distinguish viable bacteria from total population (Nocker et al., 2006). RNA-targeting techniques are an alternative way to quantify viable bacteria (Lahtinen et al., 2008). However, this method is difficult due to the short half-life of microbial RNA (Sohier et al., 2014). Molecular biology protocols require delicate steps (nucleic acids extraction and amplification) that can be tedious. Such methods are very useful for research or clinical purposes but are less suitable for quality control and industrial production settings.

Recently FC was described for absolute and specific quantification of viable and non-viable micro-organisms in multi-strain probiotic products (Chiron et al., 2018). In this study, Chiron et al. (2018) developed customized polyclonal antibodies against single probiotic strains. Evaluation of specificity confirmed that all antibodies were specific at least at the subspecies level. The authors developed a FC protocol combining these specific antibodies and membrane integrity-based viability assessment (International Standards Organisation [ISO] and International Dairy Federation, 2015). This protocol was successfully applied on commercial products containing blended probiotic strains allowing specific quantification of each strain as well as distinction of the viable and non-viable microbial populations (Chiron et al., 2018). These protocols delivered results in a very short time (<2 h) compared with classical techniques (>48 h), bringing efficient tools for research and development and quality control.

## Purity

Purity of probiotic products is especially challenging since microbiological contaminants must be detected in a background of high microbial diversity. In the United States, dietary supplement manufacturers are responsible (United States Congress, 1994) for using current GMPs (United States Food and Drug Administration, 2007, 2010) to ensure the safety and quality of their products. But these GMPs do not specify the type and level of contaminants that must assayed. Specifications tend to be regional and changing, requiring diligence to maintain marketplace compliance.

Equally challenging is the selection of analysis methods. Manufacturers use methods that are effective in a wide variety of matrices and able to detect small numbers of contaminants in the presence of large amounts of interfering substances present in samples. The preamble to the U.S. regulation 21 CFR Part 111 notes that “…you may use validated methods that can be found in official references, such as AOAC International…, USP, and others” (United States Food and Drug Administration, 2007). In acknowledgment of this official reference, the USP EP suggests that manufacturers consult materials developed by AOAC and USP early in the process of developing testing programs. The EP further suggests monitoring the FDA website for comments, warning letters, or guidance documents regarding regulatory compliance for probiotic ingredients and products in the United States.

Microbiological contaminants include pathogens, indicator organisms, and microorganisms in the manufacturing environment. Not all are safety concerns, but their presence can indicate a quality problem. To meet the challenges of establishing a microbiological contaminants program, the EP suggests evaluating candidate microorganisms by formal risk assessment. A strong risk assessment program considers industry standards, emerging pathogens, opportunistic pathogens, and rate of occurrence and/or counts of unintended microorganisms in probiotics products.

Table 3 contains the EP’s recommended limits for microbiological contaminants for probiotic dietary supplements. Acceptance criteria were established by extracting microbial contaminant data from FDA GRAS submissions for probiotics. A version of Table 3 will appear in USP General Chapter <64> *Probiotic Testing* (United States Pharmacopeia, 2019). Until implementation, the *Contaminants* criteria in pertinent Food Chemical Codex monographs should be followed.

**Table 3:** Microbiological testing and acceptance criteria for probiotic ingredients or finished products intended for oral use.

Probiotic strain category	Test	Method	Acceptance criteria (CFU/g)
**Contaminant microorganisms**			
Non-spore-forming	Non-lactic acid bacteria	ISO 13559/ IDF 153	Not more than 5 × 10^3^
	Total yeasts and molds	USP <2021>	Not more than 100
Spore-forming	Total yeasts and molds		Not more than 100
Yeasts and molds	Total aerobic microbial count		Not more than 1 × 10^3^
**Specified microorganisms**			
Non-spore-forming, spore-forming, yeasts and molds	*Escherichia coli*	USP <2022>	None detected in 10 g
	*Salmonella* spp.		

Based on low occurrence rates in probiotic ingredients and products, the EP did not include *Listeria monocytogenes, Staphylococcus aureus*, or *Pseudomonas aeruginosa* as specified microorganisms of concern. However, these species cause human illness, can occur in food manufacturing and hospital environments, and include strains known to harbor antibiotic resistances. Therefore, qualitative testing should be conducted for these microbes unless a formal risk assessment has shown that their associated risks to a probiotic ingredient does not exist or will be managed during production of the product. A formal risk assessment entails a search of literature for relevant information about a pathogen’s risk combined with review of all evaluations of ingredients through processes that impact the ingredients and products. A formal risk assessment must also be documented. Hazard Analysis and Risk Based Preventive Controls lead manufacturer’s through this process (United States Food and Drug Administration, 1997). If the risk assessment does not produce definitive information to warrant elimination of presence/absence testing for *L. monocytogenes, S. aureus*, or *P. aeruginosa*, it is best to conduct testing and gather sufficient data (history) to support a decision to continue or eliminate testing of these microorganisms. Text to support these actions will be found in General Chapter <64> (United States Pharmacopeia, 2019).

Extra scrutiny is required to ensure the safety of probiotic ingredients or products intended for at-risk populations (Sanders et al., 2016). For example, when products are targeted for infants or other immune-compromised populations, it is highly likely that risk assessment results will indicate that testing for *Clostridium perfringens* and *Cronobacter sakazakii* should be conducted. These microorganisms present a higher risk to these populations and they are present in ingredients used for infant products. In contrast, it is unlikely that the mold, *Rhizopus oryzae*, will contaminate ingredients and products intended for infant use, even though this mold was linked to an infant death (Vallabhaneni et al., 2015). In regard to *R. oryzae*, the EP suggests applying extra scrutiny during risk assessment of ingredients and products intended for infants and other at-risk populations. At present, there are no published prevalence and occurrence rates for this mold, making evidence-based risk determinations difficult.

Whether testing is prescribed in Table 3 or determined necessary through risk assessment, only appropriately validated methods established for use with probiotics should be employed. The EP strongly recommends that manufacturers ensure that their third-party laboratories use testing methods validated for probiotic samples. Two challenges are the high numbers of living microorganisms in probiotic ingredients and products and the potential for probiotic organisms to produce acid. Detection of masked targets is difficult and acids may reduce the initial target populations. Realizing the need for probiotic-suitable methods, FDA researchers (Dreher-Lesnick et al., 2015) conducted studies where low numbers of *E. coli* and *S. aureus* were detected in *Lactobacillus jensenii* cultures only after using recombinant phage lysin, LysA2, to kill probiotic cells. Further, it was reported that rapid methods [FDA Bacteriological Analytical Manual (United States Food and Drug Administration, 2018a) and United States Department of Agriculture Microbiology Laboratory Guidebook (United States Department of Agriculture, 2017)] for detection of *Salmonella* spp. and *L. monocytogenes* did not perform adequately when applied to probiotic powdered cultures (Greve et al., 2017; Shannon et al., 2017). Additionally, rapid method kit instructions needed to be modified (enrichment medium, incubation temperature, initial sample dilution) to develop and validate procedures suitable for probiotics. Combined, these studies underscore the need to determine whether standard methods are suitable for use with probiotic products and the importance of establishing whether third-party testing laboratories are using validated methods when evaluating probiotic samples.

Recently, the European Pharmacopoeia Commission announced the availability of European Pharmacopoeia 29.2, Supplement 9.7. The Supplement contains quality requirements for live biotherapeutic products intended for human use. The requirements were created to close the regulatory gap between product availability and lack of standards to ensure their quality. In current form, the standard includes one general monograph, “Live biotherapeutic products for human use (3050)” and two general chapters: “Microbial examination of live biotherapeutic products (LBP): test for enumeration of microbial contaminants (2.6.36)” and “Microbiological examination of live biotherapeutic products: test for specified microorganisms (2.6.38)”. The chapters provide methods and decision diagrams to establish method suitability (European Directorate for the Quality of Medicines and Healthcare, 2018). European Pharmacopoeia 29.2, Supplement 9.7 will be applicable in 38 countries and becomes effective 01 April 2019 (European Directorate for the Quality of Medicines and Healthcare, 2018).

## Conclusion

Driven by compelling studies showing benefit in humans, their allure as a means of improving the function of the microbiota, and a strong marketplace, probiotics are popular globally. Yet the perceived quality of commercial probiotic products suffers from a lack of transparency. Recent anecdotal information suggests that a pressing question physicians have about probiotics is whether they can trust that probiotic product labels accurately reflect what’s in the bottle. Consumers have similar concerns. Manufacturers of probiotic products range from Fortune 100 companies to small startups, from companies committed to highest quality to those willing to take shortcuts. As mentioned previously, different published assessments of probiotics have communicated that some commercial products fall short of label declarations. Although regulatory standards exist in some regions, enforcement is uneven and typically focused on safety concerns rather than accuracy in labeling. It is incumbent on probiotic manufacturers to implement comprehensive quality control programs and product design to ensure that their products meet the label claim throughout shelf-life.

We believe the time is now for industry to voluntarily improve transparency regarding probiotic product quality, even in the absence of regulatory requirements to do so. One approach is for companies to undergo unbiased third-party certification. Herein we discussed the necessary components of this process, which require collating or developing validated methods and standards for identity, quantification, and purity. Manufacturers must recognize that criteria for microbiological contaminants will become more stringent as the vulnerability of the target consumer group increases. Important scientific advances have led to new and improved approaches to some measurement challenges. WGS and PCR-based rapid techniques based on resulting DNA sequences provide accurate measures of probiotic strain identity. FC is a promising new technology that could improve efficiency of probiotic quantification. A shift to FC, however, will require a transition period, as currently CFU measurements define the dose needed for a benefit. A direct correlation between CFU and AFU for probiotics over the course of shelf-life is not available. Future clinical trials that utilize FC to quantify probiotic dose will enable FC to be used to quantify commercial products. FC has the potential to increase knowledge of physiology of probiotics used in clinical trials, which may help mechanistic investigations. Challenges remain, for example with determining composition of multi-strain blended products, and methods will need to evolve. However, the means exist today for industry to improve probiotic quality manufacturing processes and to communicate probiotic quality to end-users.

## Author Contributions

SJ, JS, CV, MP, BS, MB, and VG wrote sections of the manuscript and edited the compiled manuscript. PB wrote sections of the manuscript. JA compiled Table 1. MS wrote portions of the manuscript, compiled all author contributions, and edited to form the final version of the manuscript.

## Conflict of Interest Statement

SJ is employed by the National Institute of Standards and Technology. JS is employed by Eurofins Food Integrity and Innovation and Jean L Schoeni, LLC. CV is employed by Chr. Hansen. MP is employed by Biolab Research srl. BS is employed by DuPont Nutrition & Health. MB is employed by GNC/Nutra Manufacturing, Inc. VG is employed by US Pharmacopeial Convention. PB is owner of the business AMA Research Solutions. JA is employed by US Pharmacopeial Convention. MS reports personal fees outside the submitted work from the following entities: International Scientific Association for Probiotics and Prebiotics, Pharmavite, CD Investments, Danone USA, Danone, Yakult, California Dairy Research Foundation, Winclove BioSciences BV, Nestle, Williams Mullen, New Chapter, Dutch Mill, Clorox, Pfizer, Visalia Dairy Company, Procter & Gamble, Kelley Drye & Warren LLP, Kellogg, Trouw Nutrition, Kerry, JJHeimbach LLC, General Mills, Probi, and Medscape.

## Abbreviations

CFU: colony forming units
EP: Expert Panel
FDA: U.S. Food and Drug Administration
FC: flow cytometry
GMP: Good Manufacturing Practice
IDF: International Dairy Federation
NGS: next-generation sequencing
SNP: single nucleotide polymorphism
USP: United States Pharmacopeia
VBNC: viable but non-culturable
WGS: whole genome sequencing
